# Case Report: Malignant Melanoma Associated With COVID-19: A Coincidence or a Clue?

**DOI:** 10.3389/fmed.2022.845558

**Published:** 2022-05-26

**Authors:** A. Arturo Leis, Anna Peyton Montesi, Sariya Maryam Khan, Michael Montesi

**Affiliations:** ^1^Methodist Rehabilitation Center, Jackson, MS, United States; ^2^School of Medicine, University of Mississippi, Jackson, MS, United States; ^3^COVID Recovery Clinic, Methodist Rehabilitation Center, Jackson, MS, United States

**Keywords:** malignant melanoma (MM), COVID-19, SARS-CoV-2, cytokines, inflammation, tumorigenic

## Abstract

Following SARS-CoV-2 infection in humans, there is upregulation of proinflammatory molecules S100 calcium binding protein B (S100B), high-mobility group box-1 (HMGB1), osteopontin (OPN), tumor necrosis factor alpha (TNF-α), and other cytokines that promote hyperinflammation. The same immunoregulatory proteins that fuel the COVID-19 “cytokine storm” are also produced by melanoma cells and various other cancers to promote tumorigenesis. We report three cases of malignant melanoma (MM) associated with severe COVID-19, the first two with amelanotic melanoma and the third with hypopigmented melanoma. It is noteworthy that we did not search for these cases. Patient 1 is a personal acquaintance and cases 2 and 3 were hospitalized and worked at our rehabilitation center, respectively. We hypothesize that SARS-CoV-2 induced inflammatory tumorigenic proteins in the microenvironment that may have contributed to the *de novo* development (case 1), aggressive growth (case 2), or recurrence (case 3) of these malignant tumors. Moreover, high concentrations of the same proinflammatory proteins found in the “cytokine storm” associated with COVID-19, including TNF-α, interleukin (IL)-1α, IL-1β, IL-6, and ferritin, also induce skin depigmentation or hypopigmentation by interfering with tyrosinase synthesis, the enzyme that catalyzes the rate-limiting step of pigmentation. Hence, the marked elevation of the biological effectors that decrease skin pigmentation may also reduce pigmentation in MMs, resulting in amelanotic or hypopigmented lesions. Although it is certainly possible that the occurrence of melanoma following COVID-19 is coincidental, the ability of SARS-CoV-2 to increase expression of proinflammatory and tumorigenic molecules warrants further investigations to determine if there is an association between these disease processes or implications for patients with melanoma or other cancers who develop COVID-19.

## Introduction

Shortly after the SARS-CoV-2 pandemic began, it was recognized that an excessive inflammatory response characterized by markedly elevated inflammatory biomarkers (“cytokine storm”) contributed to the severity of COVID-19 (1–3). The significance of immunopathology in COVID-19 has since been highlighted numerous times, with results suggesting that biomarkers of hyperinflammation are strongly associated with critical illness and mortality (4, 5). On a molecular level, SARS-CoV-2 infection results in a significant upregulation of glial signaling protein S100B (6) and intracellular protein high-mobility group box-1 (HMGB1) (7, 8), two critical mediators of inflammation that are increased in human serum post-infection. The effects of S100B and HMGB1 are transduced by the receptor for advanced glycation end products (RAGE) (6–10). RAGE and its ligands play key roles in regulating the production of pro-inflammatory mediators that result in acute and chronic inflammation in various diseases with a presumed immune-mediated pathogenesis. SARS-CoV-2 infection also increases expression of osteopontin (OPN), a multifunctional cytokine that acts as a regulator of the immune response to promote inflammation and autoimmune diseases. Notably, serum OPN levels and the cleaved form of OPN have been reported to predict severity among patients with COVID-19 (11–13).

Many lines of evidence have shown that the same immunoregulatory proteins that fuel inflammation in COVID-19 are also produced by cancer cells to promote tumorigenesis. For instance, dysregulated expression of S100 proteins is a common feature of human cancers that actively contributes to tumorigenic processes such as cell proliferation, metastasis, angiogenesis, and immune evasion (14, 15). HMGB1 promotes tumor growth by enhancing proliferation and preventing cessation of cell division (cellular senescence) in cancer cells (16). It also serves as a prognostic marker in melanoma (17). RAGE signaling regulates cellular interactions during neoplastic transformation and malignant progression, including cell differentiation, proliferation, invasion and motility, resulting in increased tumor aggressiveness, and a poorer prognosis (14, 18). OPN impacts cell proliferation, survival, invasion, stem-like behavior, and also serves as a cancer biomarker (19). These findings suggest potentially strong interplay between SARS-CoV-2-induced hyperinflammation and tumorigenic cellular signaling pathways.

A foremost review on the biological, chemical, and clinical aspects of agents that play a vital role in suppressing skin pigment formation listed several key proinflammatory proteins found in high concentrations in COVID-19 immunopathogenesis as major biological effectors of depigmentation or hypopigmentation (20). These proinflammatory agents included tumor necrosis factor alpha (TNF-α), interleukin (IL)-1α, IL-1β, IL-6, and ferritin, a product of activated macrophages. At high concentrations these proinflammatory mediators induce skin depigmentation by interfering with tyrosinase synthesis, the enzyme that catalyzes the rate-limiting step of pigmentation (20). Hence, the same proteins that promote inflammation in COVID-19 and tumorigenesis in melanomas also produce depigmentation.

Here, we report three cases of pathology-proven malignant melanoma (MM) that presented after the onset of severe COVID-19. In all cases, we hypothesize that the effect of proinflammatory and tumorigenic molecular interactions following SARS-CoV-2 infection may have contributed to the *de novo* development (case 1), aggressive growth (case 2), and recurrence (case 3) of these malignant tumors. The same proinflammatory environment may also have contributed to depigmentation or hypopigmentation in these cases.

## Case 1

A 52-year-old left-handed woman of Irish descent presented in late March 2020 with fever, chills, nausea, dry cough, and frontal headache that progressed to severe flu-like symptoms with severe shortness of breath. COVID-19 was confirmed by reverse transcription polymerase chain reaction (RT-PCR) of nasal and oral specimens. Her breathing worsened and in the emergency department she was diagnosed with severe clinical COVID-19, but declined hospitalization. She was discharged home after receiving breathing treatments followed by a short course of prednisone 40 mg daily. The cough and shortness of breath persisted and she subsequently developed severe fatigue, anxiety, joint pains, alopecia, and “fog brain” with subjective memory and cognitive deficits. Four months after onset of symptoms, her partner noticed a small non-pigmented skin lesion about the size of a rounded pin head in the right upper lateral chest. The lesion continued to grow and was surgically excised in mid-October during her annual dermatology evaluation. In past years, she had several benign “wart” resections, but no skin malignancies. Both the patient and the dermatologist were surprised when a skin specimen measuring 6 × 5 × 1 mm demonstrated proliferating amelanotic abnormal melanocytes, both single cells and nests, at the dermo-epidermal junction and in the dermis with maximum Breslow’s depth 0.3 mm and Clark’s level 3. These cells were highlighted by melanoma-associated antigen recognized by T-cells (MART-1) and Sox-10, two sensitive immunohistochemical markers for the diagnosis of MM. The patient returned 2 weeks later for a wide excision of skin and underlying tissue measuring 8.5 × 2.5 × 0.6 cm that revealed focal residual melanoma *in situ* but with all margins negative. CT scan of the chest, abdomen, and pelvis with contrast was unremarkable. Three months after the onset of COVID-19 symptoms, inflammatory biomarkers and a cytokine panel (Mayo Clinic Test ID: FCYTP) were normal except for a slightly elevated C-reactive protein of 1.63 (normal <1.0 mg/dL). Immunoassays performed in October 2020 detected high levels of total IgM, IgG, and IgA antibodies to SARS-CoV-2 in the patient’s serum (59.9; normal index <1.0). As of December 2021, there has been no recurrence of the melanoma. In contrast, COVID-related “long-haulers” symptoms have persisted, including disabling fatigue, dyspnea on exertion, arthralgias, and “fog brain.”

## Case 2

**FIGURE 1:**
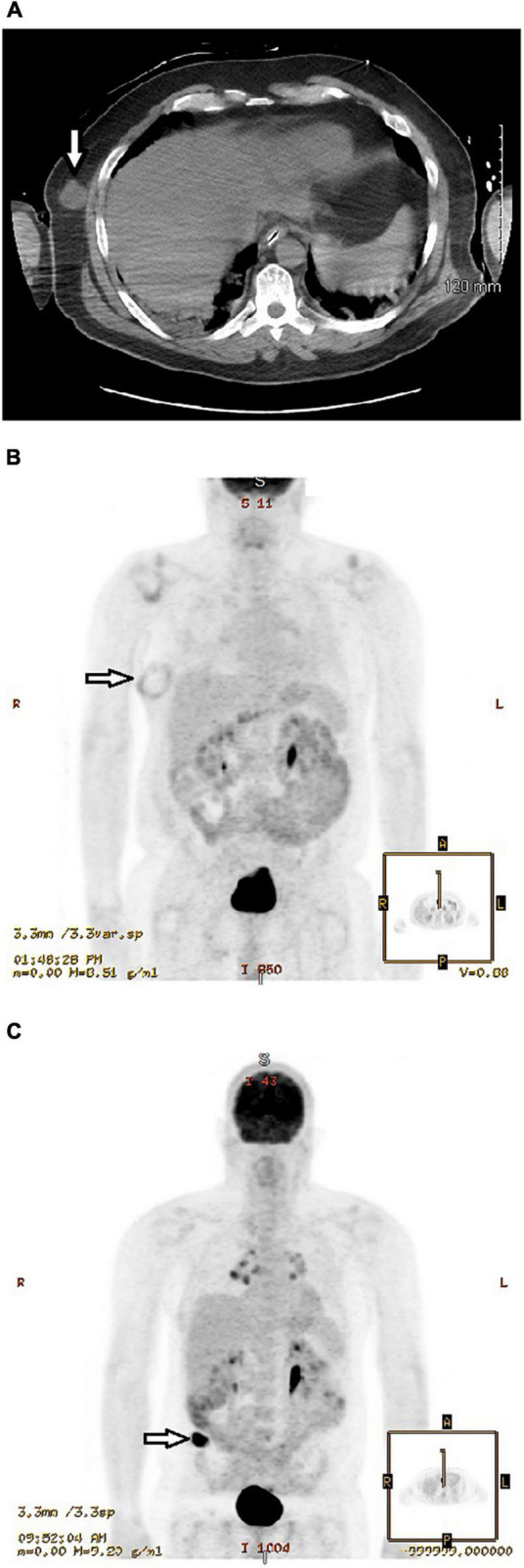
**(A)** Initial CT scan of chest without contrast performed 27 July 2020 showed extensive patchy ground-glass infiltrates throughout both lungs and an incidental 2.4 cm diameter nodular mass (arrow) in the right lateral lower chest wall of indeterminate significance and etiology. He underwent excision of the nodule on 23 October 2020. **(B)** Positron emission tomography (PET) scan performed 30 October 2020, 1 week after excision of the soft tissue nodule, showed a rounded mass that measured up to 6.7 cm with a rim of abnormal fluorodeoxyglucose (FDG) activity surrounding the mass (arrow). The enlargement was attributed in part to a hematoma that developed after the initial excision. However, a wide re-excision on 23 November 2020 showed that the melanoma had spread to the inked resection margins with positive lymph nodes containing metastatic melanoma in the subcutaneous adipose tissue and tumor nodules in the deeper subcutaneous tissue. **(C)** Positron emission tomography (PET) scan performed 27 August 2021 showed a hypermetabolic mass in the subcutaneous fat of the right buttocks region (arrow) that measured 4.7 × 2.2 cm. In comparison, PET scan on 21 May 2021, approximately 3 months earlier, showed no hypermetabolic masses or other areas of abnormal increased glucose metabolism. Biopsy of the mass confirmed malignant melanoma.

A 50-year-old physician and frontline COVID-19 hospitalist developed flu-like symptoms on 17 July 2020 and tested positive for SARS-CoV-2 by nasopharyngeal RT-PCR. He was hospitalized with hypoxemia and received the antiviral remdesivir and convalescent plasma. However, he progressed to acute respiratory distress syndrome (ARDS) requiring invasive mechanical ventilation on 26 July. Pertinent history included dermatitis controlled with chronic dapsone therapy. Laboratory studies showed elevated inflammatory biomarkers with C-reactive protein 3.9 (normal <1.0 mg/dL), ferritin 1096 (<250 ng/mL), d-dimer 10.0 (<0.4 μg/mL), and LDH 794 (100–190 U/L). Accordingly, corticosteroids (methylprednisolone 40–80 mg daily for a total of 15 days) and the immunosuppressive IL-inhibitors anakinra (an IL-1 receptor antagonist) and tocilizumab (a monoclonal antibody directed against the IL-6 receptor) were added to control hyperinflammation. Initial CT scan of the chest on July 27 showed extensive patchy ground-glass infiltrates throughout both lungs and an incidental 2.7 × 2.4 × 2.5 cm diameter soft tissue nodule in the right lateral lower chest wall (Figure 1A). The nodule was of indeterminate significance and etiology. The patient was extubated after 26 days of invasive ventilation. Thereafter, he was transferred to our rehabilitation center due to severe generalized weakness. An electromyogram (EMG) and nerve conduction studies suggested critical illness myopathy and critical illness polyneuropathy as the major causes of the weakness. The patient noticed that the mass on the right side of his chest had grown rapidly and become more pronounced following a 40-lb weight loss. He also had difficulty sleeping on his right side due to the mass and wanted it removed. Musculoskeletal examination revealed a 7 × 4 cm mass over the right chest wall, easily movable, non-tender to palpation and well circumscribed. He underwent excision of the soft tissue nodule on October 23, nearly 3-months after the initial CT scan of the chest. The pathological diagnosis was amelanotic metastatic melanoma, based on the morphologic and immunophenotypic features, including immunoreactivity for multiple melanoma-associated markers (S100, Melan-A/Mart-1, and HMB45). Positron emission tomography (PET) scan 1 week later (Figure 1B) showed a soft tissue mass that measured up to 6.7 cm with a rim of abnormal fluorodeoxyglucose (FDG) activity surrounding the mass. The enlargement was attributed in part to a hematoma that developed after the initial excision. No hypermetabolic activity was seen elsewhere. A wide re-excision measuring 8.2 × 4.7 × 2.1 cm of the initial biopsy site showed that the melanoma had spread to the inked resection margins with four positive lymph nodes containing metastatic melanoma in the subcutaneous adipose tissue and four tumor nodules in the deeper subcutaneous tissue. The rapid growth and spread of the melanoma surprised the surgeon. The melanoma tested positive for the BRAF mutation and the patient was treated with a combination of dabrafenib and trametinib, targeted therapies for the treatment of patients with advanced metastatic melanoma with BRAF mutations. Although elevated inflammatory biomarkers gradually improved with immunosuppressive therapies, 4 months after the onset of COVID-19 symptoms the ferritin level (279 ng/mL) and d-dimer (0.97 μg/mL) remained elevated and a cytokine panel showed elevations of many proinflammatory cytokines and interleukins, including TNF-α (19.5; normal ≤7.2 pg/mL), IL-1β (14.8; ≤6.7), IL-2 (3.6, ≤2.1), IL-2 receptor CD 25 soluble (979.1; range 175.3–858.2), IL-4 (4.0; ≤2.2), IL-10 (9.6; ≤2.8), and IL-13 (5.3; ≤2.8). Repeat PET scan in May 2021, showed a new hypermetabolic lesion in the right superior gluteal subcutaneous soft tissue that rapidly increased in size to 4.7 × 2.2 cm over the ensuing 2 months (Figure 1C). MRI scan of the brain in September 2021 showed a large enhancing mass in the right frontal lobe and additional smaller enhancing masses in other brain regions. Biopsies confirmed poorly differentiated metastatic melanoma. He received gamma knife radiation therapy of the brain tumors and combination immunotherapy with nivolumab and ipilimumab infusions (four cycles) to stimulate the body’s antitumor immune response. He discontinued therapy with dabrafenib and trametinib. The metastatic melanoma responded favorably to the nivolumab/ipilimumab infusions. The patient will continue to receive nivolumab monotherapy monthly for 2 years. He continues to work as an internist and in his new role as the Director of the COVID-19 Recovery Clinic at our rehabilitation center.

## Case 3

A 71-year-old woman presented in late November 2020 with nausea, severe generalized pain, arthralgias, myalgias, hoarseness of speech, worsening dyspnea and increasing confusion. In the ER, chest films showed pneumonia. Nasal RT-PCR was positive in the patient and multiple family members. Her breathing worsened and she received breathing treatments and dexamethasone. Oxygen saturation fell from 97 to 72% during sleep and she was diagnosed with COVID-related obstructive sleep apnea and placed on continuous positive airway pressure (CPAP). She also developed severe constipation with “major stomach pain,” two syncopal spells attributed to COVID-related dysautonomia, and COVID-19 “fog brain” with memory loss, “most of the illness I don’t remember.” Medical history was notable for several biopsies or excisions of skin cancers, most recently nodular basal cell carcinoma from the right lateral periorbital region in 2016 and differentiated squamous cell carcinoma in the left knee in 2014. Resections pertinent to melanoma included a junctional melanocytic nevus from the left cheek in 2006, an atypical melanocytic proliferation with hyperplasia from the left shoulder in 2011, and a superficial spreading MM of the left cheek in 2012. The greatest dimension of the cheek melanoma was 0.4 cm with maximum thickness 0.4 mm (Breslow thickness 0.4 mm) and invasion of the papillary dermis (Clark’s stage 2). She had dermatology examinations every 6 months and remained free of melanoma. Several months after developing COVID-19, she noticed a slight darkening of the area in the left cheek at the site of the previous excision. However, this was not biopsied by a dermatologist due to low clinical suspicion of MM or other malignancy. A second dermatologist, aware of the history of MM at this site, performed an excision in June 2021. The dermatologist commented that “inspection alone was not enough to diagnose MM,” but she acknowledged having “a low threshold for biopsy of any recurrent lesion in the site of a prior melanoma.” The biopsy showed proliferation of abnormal melanocytes in irregularly sized and shaped nests and confluent single-cell growth extending to peripheral and deep resection margins and down adnexal structures, as highlighted on MART-1 and SOX 10 immunohistochemical stains. The patient was informed that the recurrent invasive melanoma was “twice as deep and twice as large” as the previous malignancy, with Breslow’s depth 0.8 mm and Clark’s level 4. A nodular basal cell carcinoma measuring 4 × 3 × 1 mm was also excised from the left forehead above the eyebrow.

## Discussion

In this small case series, SARS-CoV-2 infection was a precursor to the *de novo* development of amelanotic melanoma (case 1), aggressive growth of amelanotic melanoma despite early combination therapy considered effective for treating BRAF (+) metastatic melanoma (case 2) and recurrence of MM at the site of melanoma resection 9 years earlier (case 3). It is noteworthy that we did not search for these cases; patient 1 is a personal acquaintance and cases 2 and 3 were hospitalized and worked at our rehabilitation center, respectively. In the first two cases, neither the patients nor the dermatologists expected the pathological diagnosis of amelanotic melanoma. In the last case, the classic pigmented appearance of MM may also have been lacking, since the recurrent lesion was not biopsied by the first dermatologist and a second dermatologist pursued pathological confirmation based on pertinent history rather than inspection alone. Amelanotic melanoma is a relatively uncommon form of cancer (21). Approximately 5% of melanomas are amelanotic (21). Survival after diagnosis of amelanotic melanoma is poorer than after pigmented melanoma because the lack of pigmentation removes a major identifying feature of melanomas, delaying diagnosis until a more advanced tumor stage. Moreover, the presence of mitoses on histopathological microscopic examination has been reported to be independently associated with amelanotic melanoma, suggesting that amelanotic melanomas may also grow faster than pigmented melanomas (22).

Numerous studies, ranging from clinical studies to molecular analyses, have highlighted a strong contribution of inflammation to the development of MM and other cancers. There is also evidence that the presence of inflammatory proteins in the tumor milieu, whether derived from intrinsic pathways of inflammation (melanoma cells) or extrinsic pathways (the preceding SARS-CoV-2 infection, other sources of innate immunity, genetics, tissue damage, or autoimmune disease) promote melanoma cell survival, proliferation, migration, angiogenesis, lymphangiogenesis, and invasion (14–19, 23–26). These intrinsic and extrinsic pathways of inflammation also signal the development and accumulation of myeloid-derived suppressor cells and tumor-associated macrophages, which result in T-cell tolerance and suppression of the antitumor immune response, thereby facilitating tumor growth (24, 27–29). Moreover, inflammation in the tumor microenvironment not only builds permissive conditions for melanoma progression, but also opposes cancer immunotherapy from boosting anti-tumor immunity (29).

Epidemiological studies suggest that chronic inflammation may be linked to 15–20% of cancer deaths worldwide (30, 31). Hence, the concept that virus-induced inflammation promotes tumorigenesis is not novel. In other RNA viruses, upregulation of pro-inflammatory molecules can also result in a post-infectious proinflammatory syndrome that may contribute to subacute and long-term morbidity (32–34). Following West Nile virus (WNV) infection, 44 of 140 WNV patients (31%) that reported prolonged symptoms (average symptom duration 5 years) had significantly elevated pro-inflammatory proteins (35). Moreover, an association between WNV-induced inflammation and the development and aggressive growth of primary glial neuronal tumors has been reported (36). WNV neuroinvasive disease was a precursor to the *de novo* development of a left temporal glioblastoma multiforme (GBM) that progressed to death. In a second case, malignant transformation of a relatively benign dysembryoplastic neuroepithelial tumor (DNET), a rare slow-growing supratentorial tumor associated with seizures and generally curable with surgery alone, also resulted in death (36). Such observations raise concerns that a post-infectious tumorigenic microenvironment, marked by increased concentrations of proinflammatory biomarkers and myeloid-derived cells, may promote more aggressive behavior in malignant tumors and malignant transformation in low-grade tumors. In dengue, acute cytokine release syndromes can quickly progress to hemorrhage, vascular leakage, and severe multi-organ dysfunction (formerly dengue hemorrhagic fever and dengue shock syndrome) (37), as well as persistent excessive inflammation that may persist for months or years and promote autoimmune diseases. Dengue virus infection is also associated with a higher risk of subsequently developing leukemia among adults (38). Hence, observational and population-based studies suggest that post-infectious inflammation in other RNA viruses may also promote tumorigenesis.

High concentration of several key proinflammatory proteins (ferritin, TNF-α, IL-1α, IL-1β, IL-6) induce depigmentation in normally pigmented skin by interfering with tyrosinase synthesis, the enzyme that catalyzes the rate-limiting step of pigmentation (20). These inflammatory molecules are the same proteins that are upregulated to fuel the COVID-19 “cytokine storm” and produced by melanoma cells to promote tumorigenesis. Hence, we conjectured that this inflammatory milieu could also reduce pigmentation in melanomas, resulting in amelanotic or hypomelanotic lesions. Indeed, the biochemistry of depigmentation provides a credible explanation for the amelanotic or hypomelanotic appearance (20).

## Conclusion

This small cluster of cases prompted us to hypothesize that the effect of proinflammatory and tumorigenic interactions may have played a role in the development, growth, and recurrence of these malignant tumors. While the occurrence of MM following COVID-19 may be coincidental, the same immunoregulatory molecules that are upregulated following SARS-CoV-2 infection to fuel the COVID-19 “cytokine storm” are also upregulated by melanoma cells to promote tumorigenesis. It seems reasonable to conclude that SARS-CoV-2 infection can create a microenvironment that is ideal for neoplastic transformation and malignant conversion. However, further investigation is warranted to determine if there is an association between these disease processes or implications for patients with melanoma or other cancers who develop COVID-19. The inflammatory microenvironment also provides the biological effectors that may decrease pigmentation in MMs, resulting in amelanotic or hypomelanotic appearance.

## Data Availability Statement

The raw data supporting the conclusions of this article will be made available by the authors, without undue reservation.

## Ethics Statement

The studies involving human participants were reviewed and approved by the Institutional Review Board, Methodist Rehabilitation Center, Jackson, MS, United States. Written informed consent for participation was not required for this study in accordance with the national legislation and the institutional requirements.

## Author Contributions

AL and MM contributed to conception and design of the study and wrote the final sections of the manuscript. AM and SK performed the literature searches, organized the database, and contributed to manuscript revisions. All authors read and approved the submitted version.

## Conflict of Interest

The authors declare that the research was conducted in the absence of any commercial or financial relationships that could be construed as a potential conflict of interest.

## Publisher’s Note

All claims expressed in this article are solely those of the authors and do not necessarily represent those of their affiliated organizations, or those of the publisher, the editors and the reviewers. Any product that may be evaluated in this article, or claim that may be made by its manufacturer, is not guaranteed or endorsed by the publisher.
